# Titanate Nanotubes-Based Heterogeneous Catalyst for Efficient Production of Biomass Derived Chemicals

**DOI:** 10.3389/fchem.2022.939289

**Published:** 2022-06-06

**Authors:** Shuolin Zhou, Lu Wu, Junzhuo Bai, Xianxiang Liu, Min Lei, Min Long, Keying Huang

**Affiliations:** ^1^ School of Elementary Education, Changsha Normal University, Changsha, China; ^2^ National and Local Joint Engineering Laboratory for New Petro-Chemical Materials and Fine Utilization of Resources, Key Laboratory of the Assembly and Application of Organic Functional Molecules of Hunan Province, College of Chemistry and Chemical Engineering, Hunan Normal University, Changsha, China

**Keywords:** titanate nanotubes, bio-based chemicals, catalytic reaction, supports, solid acid (base) catalyst

## Abstract

The development of efficient heterogeneous catalytic system to convert plentiful biomass to renewable bio-chemicals is urgent need. Titanate nanotubes-based materials obtained from hydrothermal treatment have been reported as low-cost and efficient catalytic materials in chemical syntheses for bio-based chemicals production with interesting catalytic performance. This mini-review expressly revealed the significance and potential of using titanate nanotubes based material as sustainable and environmentally benign solid catalysts/supports for synthesis of various bio-based chemicals, including glycerol-derived solketal, jet fuel range alkanes precursors, biomass-derived esters, aldehydes, aromatic compounds and so on. From the current knowledge on titanate nanotubes-based material via hydrothermal method here summarized, the future lines of research in the field of catalysis/supports for bio-based chemicals production are outlined.

## Introduction

At the present moment, fossil fuels are the primary sources of energy for humankind. However, the use of fossil fuels often associated with the concerns, such as price fluctuation, long-term availability, and growing environmental effects (Brockway et al., 2019). Besides, the quest for global energy and chemicals needs will be in high demand due to the rapidly developments of economic and socioeconomic. Hence, transforming renewable energy into alternative fuels and chemicals is an essential and indispensable pathway (Gielen et al., 2019; Stančin et al., 2020). Biomass is an abundant renewable and cleaner resource, which can be converted into a wide range of various fuel grade molecules and bio-chemicals as alternatives to fossil-derived products (Li et al., 2017; Schutyser et al., 2018; Okolie et al., 2021; Ashokkumar et al., 2022). In this situation, new chemical technology and efficient catalysts to convert plentiful biomass to renewable bio-chemicals is urgent need.

Titanate nanotubes (TNTs), a typical of Ti-based material, have attracted extensive researches due to its novel properties such as chemical stability, large surface area, non-toxicity, and relatively hydrophobic nature, which have shown great potential not only as catalysts but also as supports. A lot of literatures have described the synthesis of titanate nanotubes by various methods such as hydrothermal treatment (Kasuga et al., 1998; Kasuga et al., 1999), template-assisted method (Zhang et al., 2001), and the anodizing of titanium metal (Gong et al., 2001). It is worth noted that TNTs obtained by hydrothermal treatment of TiO_2_ nanoparticles in the absence of a template and at low temperature (120–150°C), which has received significant attention (Bavykin et al., 2006). In recent years, many articles and reviews have covered the applications of hydrothermally synthesized TNTs materials in photoelectrochemial reactions (Arifin et al., 2021), photocatalytic (Ji et al., 2022), dye-sensitized solar cells (Madurai Ramakrishnan et al., 2020; Souza et al., 2021), adsorbents (Li et al., 2021) and other interesting applications (Yao et al., 2020). In addition, the structural, optical, thermal and morphological properties of TNTs synthesized by conventional hydrothermal method were systematically discussed in other reviews (Ou and Lo, 2007; Muniyappan et al., 2017; Rempel et al., 2021).

**FIGURE 1:**
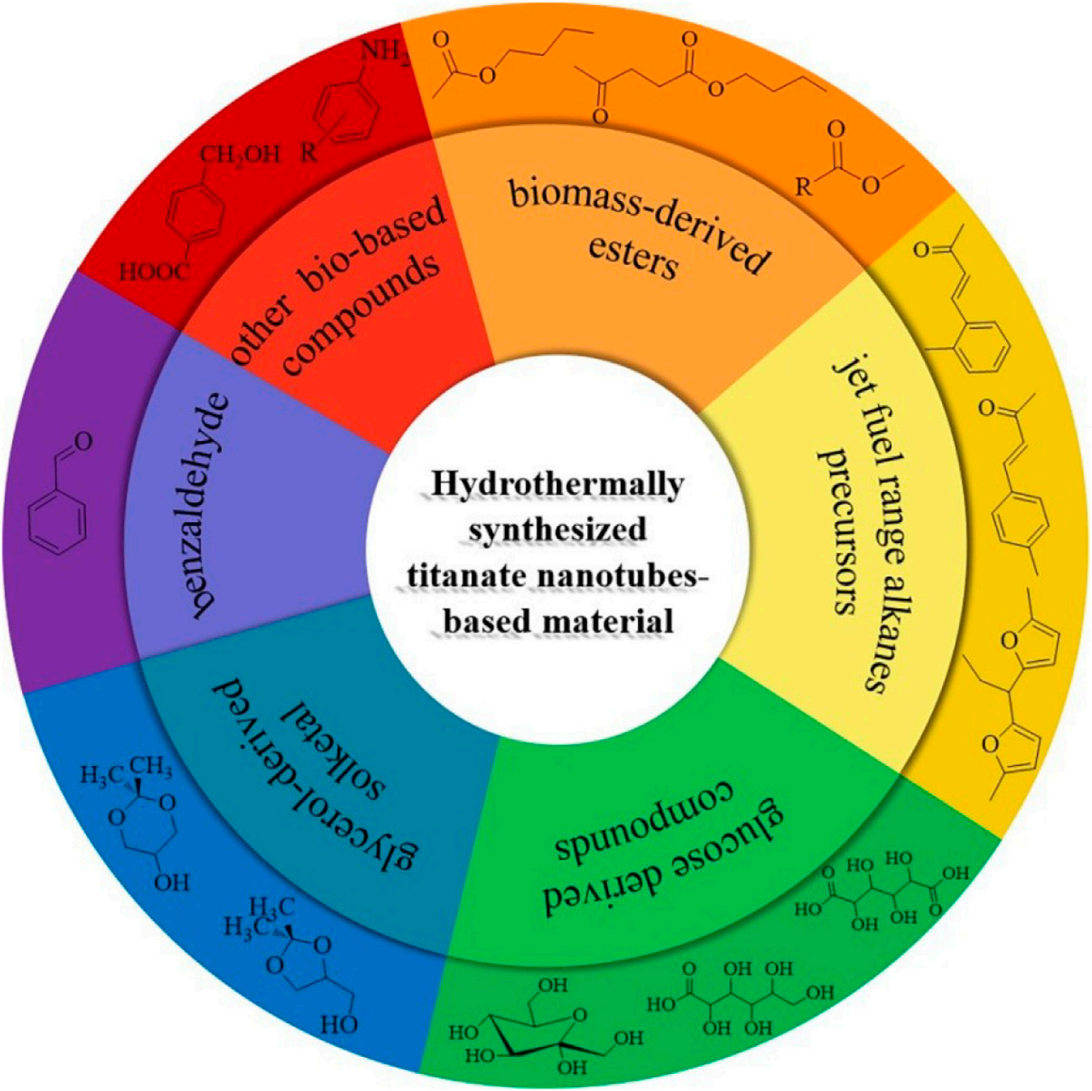
The schematic of production of typical biomass derived chemicals over titanate nanotubes-based catalyst.

Kitano and co-workers found that the TNTs exhibited excellent catalytic performance in Friedel-Crafts alkylation and the 5-hydroxymethylfurfural production (Kitano et al., 2010). This finding may potentially open up new catalytic applications of the titanate nanotubes for organic transformations and biomass conversion. In the past few years, some studies have been reported the use of TNTs in various acid (base) catalyzed organic chemical transformation (Kitano et al., 2013; Wada et al., 2013; Li et al., 2015; Reddy et al., 2015). This mini-review focuses on TNTs based material as solid catalysts/supports with the potential application for the bio-based chemicals production (Figure 1).

## Biomass Derived Chemicals Production

### Production of Glycerol-Derived Solketal

The acetalization reaction has been widely studied because they are important and efficient processes to convert aldehydes or ketones into high-valued compounds. The glycerol-derived solketal as oxygenated compound may be incorporate into additive for standard diesel fuel as well as decrease the emission of hydrocarbons and particulate species (Talebian-Kiakalaieh et al., 2018). De Carvalho et al. reported the acetalization of glycerol using the protonated titanate nanotubes as a solid catalyst (de Carvalho et al., 2017). The synthesis time of TNTs and the role of structure on the catalytic performance were systematically investigated. The best performance towards glycerol conversion was achieved by the TNTs synthesized at 72 h with high textural, morphological and acidity properties. The glycerol conversion was 44.4% and the selectivity toward the desired products (solketal and acetal) was observed to be more than 98% at 50°C and an acetone/glycerol molar ratio of 1. Decoration of transition and rare earth metal nanoparticles on titanate nanotubes has a considerable effect on the number of Brønsted and Lewis acidity sites (Camposeco et al., 2016; Wada et al., 2016). These researches demonstrated that various metals (Ag, Au, Ce, Fe, Mn, Pd, Pt, V, W and Nb) obviously improve the Brønsted acidity of TNTs except for La; some metals such as Pt, Mn and W could enhance Lewis acidity of TNTs; the addition of transition metals (V, Mn, W and Fe) increased remarkably the total acidity of TNTs. In another study, TNTs incorporated with metals (Pt, Co, Ni) were synthesized and applied in acetalization of glycerol with acetone (Gomes et al., 2018). Interestingly, the Pt-containing titanate nanotubes (Pt-TNT) catalyst has high catalytic activity, affording 46.7% of glycerol conversion with 10% selectivity to solketal, whereas the Co and Ni-incorporated titanate nanotubes exhibited relativity low catalytic activity (glycerol conversion <5%). Structural properties of various titanate nanotubes-based acid catalysts are listed Table 1. The suitable tuning of pore-structure and proper surface acidity of Pt-TNT were contributed to the resulting in a stable solid for this reaction (Gomes et al., 2018). It is noteworthy that leaching of Co and Ni species is reported as the main mechanism for the catalyst deactivation for Co-containing titanate nanotubes and Ni-containing titanate nanotubes. This demonstrated that the catalytic activity and recyclability of TNTs could be tuned by incorporation of a suitable metal.

**TABLE 1:** Structural properties of various titanate nanotubes-based acid catalysts.

Catalyst	S_BET_ (m^2^/g)	Pore Volume (cm^3^/g)	Mesopore Diameter (nm)	Acidity (mmol/g)	Ref.
HTNT48	313	0.81	9.0	0.24	de Carvalho et al. (2017)
HTNT72	182	0.74	11.5	0.33	de Carvalho et al. (2017)
Co-TNT	236	0.84	11.8	0.093	Coelho et al. (2016); Gomes et al. (2018)
Ni-TNT	222	0.84	12.6	0.103	Coelho et al. (2016); Gomes et al. (2018)
Pt-TNT	182	0.64	12.2	0.261	Coelho et al. (2016); Gomes et al. (2018)

### Jet Fuel Range Alkanes Precursors Synthesis

Aldol condensation approach is the very important for synthesis of high-quality bio-fuels (He et al., 2021). Protonated titanate nanostructures used as solid acid catalysts exhibited the excellent catalytic activity in the condensation reaction between various benzaldehyde derivatives and cyclohexanone (Sluban et al., 2017). A higher conversion rate was obtained over TNTs as compared to titanate nanoribbons, demonstrating the beneficial role of nanotube morphology. The catalyst showed a remarkable stability since no significant decrease in the catalytic activity in five cycles. In addition, protonated titanate nanotubes did not require any activation prior to the reaction. Recently, protonated titanate nanotubes catalyst displayed much higher activity for acid-catalyzed aldol condensation of methyl benzaldehyde and acetone, two platform compounds obtained from lignocelluloses (Timothy et al., 2020). Around 76% yield of jet fuel precursors, namely 4-(o-tolyl)but-3-en-2-one, was obtained under the optimum reaction conditions. After the hydrodeoxygenation (HDO) of jet fuel precursors in cyclohexane under mild conditions (403K, 5MPa, 2 h), high yields ( ∼ 90%) of dicycloalkanes were achieved. They discovered the catalytic activity of protonated titanate nanotubes was higher than TiO_2_ P25 and titanate nanowire under the same conditions. It is suggested that the special nanotube morphology, bigger surface area, higher acid site amount and acid strength could be considered as the reasons for the good catalytic performance of protonated titanate nanotube. It is also found that the protonated titanate nanotubes catalyst was stable and could be repeatedly used for five runs without significant deactivation.

The protonated titanate nanotubes as a good solid catalyst was applied for the hydroxyalkylation/alkylation (HAA) of 2-methylfuran (2-MF) with *n*-butanal from lignocelluloses to synthesize diesel and jet fuel range alkanes precursors. Compared to other inorganic solid acids such as SO_4_^2-^/ZrO_2_, ZrP and H-ZSM-5, protonated titanate nanotubes has higher catalytic activity, giving 77% yield of HAA product under mild reaction conditions (Li et al., 2015). The protonated titanate nanotubes was also effective for the catalytic HAA of 2-MF with other lignocellulosic carbonyl compounds, such as furfural, acetone and mesityl oxide. The outstanding catalytic performance of protonated titanate nanotubes for the HAA of 2-MF and n-butanal can be explained by the following reasons: 1) the protonated titanate nanotubes has higher specific surface area, which is beneficial for the adsorption of reactants, 2) the transformation of commercial TiO_2_ P25 to protonated titanate nanotubes leads to the higher acidity (the amount of acid sites and the generation of strong acid sites), and 3) the generation of Brönsted acid sites may be beneficial to the HAA reaction of 2-MF and n-butanal.

### Preparation of Biomass-Derived Esters

Some researchers have suggested that the sodium titanate nanotubes is an effcient heterogenous base catalyst in the transesterification reactions, which is the most common route for biodiesel production (Hernández-Hipólito et al., 2014). Recently, the sodium titanates were used as catalysts in the transesterification of pure and cooked oils into biodiesel (Zaki et al., 2019). The biodiesel yield was found to be 95.9% at 80°C for 2 h; the authors discovered that the catalyst showed high activity for cooked oil conversion, with yields of 96.0, 96.0, and 93.58% for the first, second, and third uses of oil, respectively. The authors found that the transesterification reaction preferentially proceeded via dual-site Langmuir-Hinshelwood mechanism with the aid of the Density Functional Theory (DFT), Monte Carlo (MC) simulation, and molecular dynamics simulation. Furthermore, the transesterification reaction kinetics followed a pseudo-first-order kinetics model. Simiarlly, the sodium titanate catalysts were prepared by sol–gel hydrothermal method, and the synthesis parameters of sodium titanates on the catalysts activity in soybean oil conversion to biodiesel were discussed using a factorial design (Machorro López et al., 2021). Combing the characterization results and catalytic results, the authors pointed out that trititanate was the most efficient in the conversion of soybean oil to biodiesel, achieving around 80% conversion. Doping metal ions on sodium titanate nanotubes may be an important strategy to improve the catalytic acitivity. For example, sodium titanate nanotubes doped with potassium proved as a efficient catalyst for transesterification of soybean oil with methanol (Hernández-Hipólito et al., 2015; Martínez-Klimova et al., 2016). Recently, the promoting role of sodium carbonate addition to sodium titanate nanotubes were reported (Martínez-Klimov et al., 2020). Incorporation of Na_2_CO_3_ (3–10 wt%) to sodium titanate nanotubes can increase the amount of strong basic sites in the catalysts. A synergetic effect between Na_2_CO_3_ and sodium titanate nanotubes was proposed for the increase in the amount of strong basic sites, resulting in an excellent catalytic performance in transformation of triglycerides to methyl esters (97% yield). Interestingly, in other important studies, lipase immobilized onto the sodium titanate nanotubes have recently been employed in the fatty acid methyl esters production (Nady et al., 2020; El-Kady et al., 2021). The immobilized lipase gave a high fatty acid methyl esters yield of 83.5% at short time of 90 min and showed the enhanced recycling stability for ten consecutive cycles.

Esterification is the most common reaction for biomass conversion and high-valued chemicals production (Zhang et al., 2019). Xu et al. found the catalytic performance of the titanate nanotubes is significantly higher than titanate nanosheets and layered H_2_TiO_7_ in esterification of acetic acid with n-butanol (Xu et al., 2020). The authors proposed that the surface acid characteristics and confinement effect were responsible for the high catalytic activity of titanate nanotubes. This clearly reveals that the microstructure is important to the catalytic activity. The finite amount of catalytic sites on the TNTs, however, would hamper in practical applications. It is noteworthy that TNTs prepared by hydrothermal method with abundant hydroxyl groups. Thus, the potential to modify TiO_2_ nanotubes to incorporate organosulfonic acid groups open new perspectives for their use as solid acid catalysts in a variety of reactions. Our groups reported various titanate nanotubes-bonded organosulfonic acid catalysts for the esterification of biomass-derived levulinic acid with n-butyl alcohol (Zhou et al., 2018; Zhou et al., 2019; Zhou et al., 2022a). Up to 98.9% yield of *n*-butyl levulinate was obtained under the optimal reaction conditions. In these hybrid catalysts the acid sites are covalently linked on titanate nanoutbes, therefore, they showed an excellent reusability with a slight decrease in several runs. On the other hand, the incorporation of organic groups on the TNTs can tune surface hydrophobicity property. Recently, in order to recycle heterogeneous acid catalysts from the reaction mixture, a new solid acid catalyst Fe_3_O_4_@TNTs-SO_3_H was successfully synthesized and applied to esterification of renewable levulinic acid to fuel additive n-butyl levulinate (Mao et al., 2020). This catalyst was demonstrated to show high catalytic activity, affording n-butyl levulinate with a yield of 94.6% under optimum conditions; the catalyst could be reused for 6 times. It is believed that titanate nanotubes can be rationally designed via post-synthesis strategy to prepare solid acid catalysts with excellent performance.

The alcoholysis process has been reported as a highly reactive method for conversion of lignocellulose to valuable chemicals (Zhu et al., 2017). Sulfonic acid functionalized TiO_2_ nanotubes were prepared by the sulphonation reaction of hydrothermally synthesized Titanate nanotubes using chlorosulfonic acid as the sulfating agent in our recent work (Zhou et al., 2022b). About 79.9% yield of *n*-butyl levulinate was achieved in the alcoholysis of the furfuryl alcohol with n-butanol under mild conditions. In addition, the catalysts showed a stable catalytic performance after four consecutive cycles. The covalently linked –SO_3_H groups on the TNTs surface was responsible for the stability of catalyst.

### Synthesis of Glucose Derived Compounds

Kumar and co-workers have recently reported a sodium titanate nanotubes as a potential Lewis base catalyst for large-scale demonstration of glucose isomerization to fructose in aqueous media (Kumar et al., 2018). In this work, the glucose conversion could be reached with 31.26% fructose yield and 65.26% selectivity under relatively lower operating conditions for 15 min or less. They found that the presence of large basic sites in sodium titanate nanotubes was contribute to the higher glucose conversion. Additionally, the catalyst could be effciently recycled and regenerated by a simple NaOH treatment. On the contray, protonated titanate nanotubes was reported as solid acid catalyst for conversion of glucose into HMF via isomerization and dehydration process, giving the moderate yield of HMF (Kitano et al., 2010). Recently, protonated titanate nanotubes exhibit relatively high catalytic performance for isomerizaiton of alpha pinene, an inexpensive and important essential oil which is widely used in the synthesis of various fine chemicals (Huang et al., 2020). Hence, it is believed that the protonated titanate nanotubes/sodium titanate nanotubes can be uesed as acid or base catalyst in different types of isomerization reactions for bio-based chemicals production.

More importantly, TNTs are regarded as an attractive support material because they exhibit large surface area, high surface hydroxyl density, high ion-exchange capacity and the good stability. Recently, the catalytic performance of Au-Pd nanoparticles prepared by colloidal synthesis and immobilised on titanate nanotubes in the selective oxidation of glucose to gluconic and glucaric acids has been studied by Khawaji et al. under relatively mild conditions (Khawaji et al., 2019). They found that Au-rich catalysts favored deep oxidation to glucaric acid while Pd-rich catalysts displayed the formation of gluconic acid. It is suggested that the bimetallic composition of Au and Pd on TNTs could be tuned to enhance the production of either gluconic acid or glucaric acid.

### Selective Oxidation of Benzyl Alcohol to Benzaldehyde

The selective oxidation is potentially key reaction in the biomass conversion and value-added chemicals production (Nasrollahzadeh et al., 2020). The selective oxidation of benzyl alcohol, a typical biomass derivative, to corresponding carbonyl compounds has been received much attention. A highly active Au-Pd on titanate nanotubes (Au-Pd/Ti-NT) catalyst has been produced by using colloidal synthesis and immobilisation on sodium-free Ti-nanotubes (Khawaji and Chadwick, 2017). The catalyst has markedly superior catalytic activity (turn over frequency>19 ,000 h^−1^) for the selective oxidation of benzyl alcohol compared with similar catalysts reported in the literature such as Au-Pd catalysts supported on Ti-NTs prepared by adsorption as well as conventional Au-Pd/TiO_2_ prepared by impregnation. The authors claimed that the superior catalytic activity of the catalyst is attributed to the high metal dispersion on the external surfaces of titanate nanotubes, the narrow particle size distribution, and the high degree of Au-Pd mixed alloying. Moreover, the effect of the catalyst preparation method on the selective oxidation catalytic activity of Au-Pd supported on titanate nanotubes (Au-Pd/Ti-NT) was further investigated (Khawaji and Chadwick, 2019). The most active Au-Pd/Ti-NT catalyst for the selective oxidation of benzyl alcohol is shown to be that prepared using colloidal synthesis and immobilization with PVA as a stabilizer, which has markedly superior catalytic activity compared to catalysts prepared by deposition-precipitation, adsorption, and dry impregnation methods. Therefore, it is very importance to select a synthesis method to obtain optimal catalytic performance. Besides, the morphology and physiochemical properties of the support were also found to play a crucial role for catalytic oxidation activity, selectivity, and stability (Khawaji and Chadwick, 2018; Khawaji and Chadwick, 2020). Furthermore, exploring the utilization of TNTs-based material as a photocatalyst for selective oxidation of benzyl alcohol will be desirable under ambient conditions (Yang et al., 2016).

### Production of Other Bio-Based Chemicals

Hydrogenation reactions are considered as valuable and key technologies in biomass conversion processes (Li et al., 2019). Titanate nanotubes supported Pd was applied to hydrogenation of 4-carboxy-benzaldehyde, displaying a better catalytic performance than the commercial Pd/C catalyst (Liu et al., 2018). Meanwhile, Torres’ group reported the selective hydrogenation of nitrobenzenes over gold nanoparticles supported on titania nanotubes in liquid phase at room temperature (Torres et al., 2018). It was found that the selectivity towards p-substituted anilines reached 90% for all substrates in their study. Recently, other nobel metals, such Pt and Pd, confined on titanate nanotubes also performed well for the hydrogenation of nitroarenes and other substituted-nitroarenes (Shanmugaraj et al., 2022). These works indicated that the TNTs has the hollow tubular structure, the abundant –OH groups and strong metal–support interaction which renders them excellent supports for preparing TNT-supported catalysts for hydrogenation of various compounds to high-value chemicals.

Transforming of CO_2_ conversion into hydrocarbons recently has received significant attention (Díaz de León et al., 2019). A ternary hybrid catalyst, poly (ethyleneimine)-tethered Ir complex catalyst immobilized in titanate nanotubes were applied to hydrogenation of CO_2_ to formic acid under the relatively mild conditions (Kuwahara et al., 2017). Kuwahara et al. stated that the ability of TNTs to efficiently capture CO_2_ and to stabilize PEI, where Na^+^-type TNTs with higher basic property provides more productive effect, which are responsible for the high catalytic performances. Recently, the catalytic activity of rhodium supported on titanate nanotubes was evaluated by *in situ* infrared study in the synthesis of formic acid *via* CO_2_ hydrogenation (Ruiz-García et al., 2019). For this catalyst a turn over frequency (TOF) of 7.2 × 10^−2^ h^−1^ was obtained at 90°C and atmospheric pressure. Furthermore, the authors provided the evidence of active surface species bonded to support sites and to rhodium sites via *in-situ* studies. Besides, photocataytic CO_2_ conversion to hydrocarbon fuel using TiO_2_ based material is another important strategy (Razzaq and In, 2019).

## Conclusion

In summary, titanate nanotubes-based heterogeneous catalyst prepared via hydrothermal method have been critically outlined and discussed in this mini-review as a promising catalyst/support for bio-based chemicals production. Titanate nanotubes can be modified by a variety of metal or non-metal dopants or be functionalized by organic surface modification to increase the acid and/or base properties of titanate nanotubes, thereby enhancing the catalytic activity and selectivity. Their very interesting properties make them promising catalysts for use in various reactions, such as acetalization/condensation, hydroxyalkylation/alkylation, transesterification, esterification, alcoholysis and isomerization for production of biomass derived chemicals. The described titanate nanotubes-based heterogeneous catalyst with different catalytic properties can be utilized as low-cost, efficient, sustainable and versatile materials. Enlightened by the hollow tubular structure, confinement effect and strong metal–support interaction, much works on the rational design of multifunctional catalyst for selective oxidation and hydrogenation reaction are ongoing. Although these catalysts have some advantages such as simple separation, and recycling, it would be highly desirable to keep the catalysts intrinsic characteristics that will enhance the catalytic stability of titanate nanotubes-based material under extreme environment. Therefore, except for traditional catalyst characterization, a combination of *in situ* catalytic studies and theoretical calculations and simulations are also helpful to provide valuable information toward the structure-activity relationships of titanate nanotubes-based catalysts. This review is also expected to act as a key reference to researchers for developing advanced titanate nanotubes-based catalysts in large scale applications for bio-based chemicals production with resulting in significant developments.
